# Role of N-Terminal His_6_-Tags in Binding and Efficient Translocation of Polypeptides into Cells Using Anthrax Protective Antigen (PA)

**DOI:** 10.1371/journal.pone.0046964

**Published:** 2012-10-08

**Authors:** Christoph Beitzinger, Caroline Stefani, Angelika Kronhardt, Monica Rolando, Gilles Flatau, Emmanuel Lemichez, Roland Benz

**Affiliations:** 1 Rudolf-Virchow-Center, DFG-Research Center for Experimental Biomedicine, University of Würzburg, Würzburg, Germany; 2 Toxines microbiennes dans la relation hôte-pathogènes, C3M, U1065, Inserm, Nice, France; 3 UFR Médecine, IFR50, Université de Nice-Sophia Antipolis, Nice, France; 4 School of Engineering and Science, Jacobs University Bremen, Bremen, Germany; Universidad Nacional Autonoma de Mexico, Instituto de Biotecnologia, Mexico

## Abstract

It is of interest to define bacterial toxin biochemical properties to use them as molecular-syringe devices in order to deliver enzymatic activities into host cells. Binary toxins of the AB_7/8_-type are among the most potent and specialized bacterial protein toxins. The B subunits oligomerize to form a pore that binds with high affinity host cell receptors and the enzymatic A subunit. This allows the endocytosis of the complex and subsequent injection of the A subunit into the cytosol of the host cells. Here we report that the addition of an N-terminal His_6_-tag to different proteins increased their binding affinity to the protective antigen (PA) PA_63_-channels, irrespective if they are related (C2I) or unrelated (gpJ, EDIN) to the AB_7/8_-family of toxins. His_6_-EDIN exhibited voltage-dependent increase of the stability constant for binding by a factor of about 25 when the *trans*-side corresponding to the cell interior was set to −70 mV. Surprisingly, the *C. botulinum* toxin C2II-channel did not share this feature of PA_63_. Cell-based experiments demonstrated that addition of an N-terminal His_6_-tag promoted also intoxication of endothelial cells by C2I or EDIN via PA_63_. Our results revealed that addition of His_6_-tags to several factors increase their binding properties to PA_63_ and enhance the property to intoxicate cells.

## Introduction

Gram-positive bacteria such as *Bacillus anthracis* and *Clostridium botulinum* synthesize as most crucial virulence factors protein toxins of the AB_7/8_ type. These toxins are composed of two components which are nontoxic by themselves when added to the external media of target cells [1]. One or more A-components of the toxins feature intracellular enzymatic activity and are responsible for the toxicity. The B-component binds to cellular receptors or directly to the membrane and transports the enzymatic component into the cell. Anthrax-toxin from *B. anthracis* belongs to the AB_7/8_-type of toxins classified by a pore forming B-component, protective antigen (PA) and two enzymes, edema factor (EF) and lethal factor (LF). PA is an 83 kDa water soluble precursor, which has to be activated by cleavage of a 20 kDa N-terminal part to form the functional PA_63_-heptamers/octamers [2]–[5]. The proteolytic activation is performed *in vivo* by cell bound furin. It allows pore formation and injection of both enzymatic components EF and LF into cells [6]–[9]. EF is an 89 kDa Ca^2+^- and calmodulin-dependent adenylate cyclase which causes severe edema by uncontrolled increase of the intracellular concentration of cAMP. LF is a Zn^2+^-binding metalloprotease that cleaves mitogen-activated protein kinase kinases (MAPK-kinases).

*C. botulinum,* well known for the production of potent neurotoxins, also produces other protein toxins such as the binary C2-toxin and the single-component C3 exoenzyme [10]–[12]. The homologous pore forming component to PA_63_ of C2-toxin is C2II. After proteolytic cleavage with trypsin (60 kDa) it forms heptamer/octamerss that insert into biological and artificial membranes at an acidic pH and promotes the translocation of the 45 kDa enzymatic component C2I. Similar to anthrax-toxin a receptor-mediated endocytocis of C2 is required for intoxication of the cell [13], [14]. C2I acts as an ADP-ribosyltransferase on arginine177 of monomeric G-actin, causing disruption of the actin cytoskeleton of eukaryotic cells [15], [16].

The toxins of the AB-type represent simple but sophisticated molecular syringes for protein delivery into target cells. This means that they could be important systems for development of new strategies for efficient injection of polypeptides into target cells. Possible Trojan Horses could be binary toxins of the AB_7/8_ type such as anthrax- and C2-toxin [1]. The binding of the N-terminal ends of the enzymatic components to the heptameric/octameric channel formed by the binding components is followed by receptor-mediated endocytosis, acidification of the endosomes and final release of the enzymatic components into the cytosol of target cells, where they exert their enzymatic activities [3], [17]. Interestingly, the amino-terminal part of LF is sufficient to confer the ability to associate with PA_63_-heptamer/octamerss on LF. It can be used to drive the translocation of unrelated polypeptides fused to LF_1–254_ into target cells in a PA_63_-dependent manner [18], [19]. Although the enzymatic components of anthrax- and C2-toxin differ considerably in their enzymatic activity and in their primary structures as well, the binding components PA and C2II share a significant overall sequence homology of about 35%, which means that they are closely related in structure and probably also in function [5], [8], [20], [21]. There exist several structural features that are important for the binding of channel blockers and enzymes to be delivered into the target cells. One is the so-called Φ(phenylalanine)-clamp - F427 in PA and F428 in C2II – which is combined with two rings of seven negatively charged amino acids - E399 and D428 in PA and E398 and D427 in C2II, respectively [22], [23]. These negatively charged amino acids seem to interact with the positively charged N-terminal ends of the enzymatic components [19], [24], [25]. Another interesting feature involved in binding and transport of truncated and full-length effectors is the alpha-clamp in PA [26], [27]. This represents a big amphipathic cleft on the surface of the PA_63_ heptamers/octamers, which plays an important role in oligomer formation of PA_63_ and unfolding and translocation of effectors [26], [27].

Blanke et al. [19] have established that addition of His_6_-tag and other polycationic presequences to diphtheria toxin A chain (DTA) allows its injection into cells by PA_63_ binding component. The observation that polycationic peptides at the N-terminal end of DTA facilitate its import into the cytosol of a CHO-K1 cell line is of particular interest [19]. Here we aimed at establishing whether this effect of His_6_-tag could be generalized to other toxin enzymatic components and quantify the involvement of His_6_-tag on these toxin component interactions with PA_63_ in vivo and in vitro. To study this we have investigated the influence of additional charges on the N-terminal end on binding of the enzymatic factors to the channels formed by PA_63_ and C2II. First results in the field were found with polycationic peptides fused to EF, LF, LF_N_, EF_N_ and DTA [19], [21]. Our results suggested that the binding of LF and EF to C2II is possible and that C2I binds to PA_63_ in the black lipid bilayer assay as well. The most significant result that was observed was a preferential binding of His_6_-C2I, as compared to C2I, to PA_63_. Interestingly, PA_63_ is able to transport His6-C2I into target cells with a higher efficiency than C2I. We extended these finding by demonstrating that a His_6_-tag fused to the N-terminal end of the epidermal cell differentiation inhibitor (EDIN) of *Staphylococcus aureus* also increases its affinity to PA_63_ and allows an efficient PA_63_-dependent delivery of His_6_-EDIN in target cells. In addition, the stability constant for binding of His_6_-EDIN and not that of EDIN to PA_63_-channels was found to be highly voltage-dependent, which could be one important factor for efficient delivery of effectors via PA_63_ into target cells.

## Experimental Procedures

### Materials

Protective antigen encoding gene was cloned with *Bam*HI-*Sac*I restriction sites into pET22 (Novagen) as previously described [28]. The translocation-defective PA mutant F427A [22], [29] was constructed by site-directed mutagenesis using the QuickChange™ kit (Stratagene) according to the manufactureŕs instructions. The PA-gene cloned in the plasmid pET19 (Novagen) [30], [31], was used as a template. The construct was confirmed by DNA sequencing. The protein was expressed with an N-terminal His_6_-tag in BL21 (DE3) (Novagen) and purified by HiTrap chelating (Pharmacia) charged with Ni^2+^ ions.

C2I and C2II genes were PCR-amplified from genomic DNA of *Clostridium botulinum* D strain 1873 and cloned into pET22 (Novagen) and pQE30 (Qiagen) expression plasmids with *Bam*HI-*Sac*I restriction sites.

The plasmid coding for the chimera protein MBP-gpJ (maltose-binding-protein attached to amino acids 684–1132 of Lambda phage tail protein J) was a kind gift of Alain Charbit, Necker Enfants Malade, Paris, France. Expression and purification of MBP-gpJ was performed as described previously [32]. gpJ was obtained by treatment of MBP-gpJ bound to starch column beads (amylose-Sepharose, New England Biolabs) with factor X_a_ (Invitrogen). His_6_-gpJ (684–1132) was obtained as described previously [33].

The DNA encoding EDIN (NCBI M63917) was cloned into pET28a vector using *Bam*HI*-Eco*RI restriction site as described previously [34]. Recombinant toxins containing His_6_-tags were expressed in *E. coli* BL21 (DE3) and purified on a Chelating Sepharose Fast Flow column previously chelated with nickel (Amersham Biosciences) as recommended by the manufacturer. Fractions containing toxin were pooled and dialyzed over night against 250 mM NaCl and 25 mM Tris-HCl, pH 8. The N-terminal His_6_-tag was removed by incubation with thrombin. Nicked anthrax PA_63_ from *B. anthracis* was obtained from List Biological Laboratories Inc., Campbell, CA. One mg of lyophilized protein was dissolved in 1 ml 5 mM HEPES, 50 mM NaCl, pH 7.5 complemented with 1.25% trehalose. Aliquots were stored at −20°C. Channel formation by PA_63_ was stable for months under these conditions.

### Cell Culture and Biochemical Products

HUVECs (human umbilical vein endothelial cells, a human primary cell line obtained from PromoCell) were grown in serum-free medium (SFM) supplemented with 20% FBS (Invitrogen), 20 ng/ml basic ßFGF (Invitrogen), 10 ng/ml EGF (Invitrogen) and 1 µg/ml heparin (Sigma-Aldrich) as described previously [35]. Monoclonal antibodies used were: anti-RhoA (BD Biosciences, [clone 26C4]); anti- ß-actin (SIGMA, [clone AC9–74]); anti-His-tag (Qiagen, [Penta-His]). Primary antibodies were visualized using goat anti-mouse horseradish peroxidase-conjugated secondary antibodies (DakoCytomation), followed by chemiluminescence detection ECL (GE Healthcare). Levels of active Rho were determined by GST-rhotekin RBD pull-down that was modified as described previously [35].

### Immunofluorescence Studies

The experiments were performed on cells fixed in 4% paraformaldehyde (SIGMA). Actin cytoskeleton was labelled using FITC-conjugated phalloidin (SIGMA), as described in [36]. Immunosignals were analyzed with inverted microscope (EVOS*fl*, AMG) using a 20X object lens.

### Lipid Bilayer Experiments

Black lipid bilayer measurements were performed as described previously [37]. The instrumentation consisted of a Teflon chamber with two aqueous compartments connected by a small circular hole. The hole had a surface area of about 0.4 mm^2^. Membranes were formed by painting a 1% solution of diphytanoyl phosphatidylcholine (Avanti Polar Lipids, Alabaster, AL) in n-decane onto the hole. The aqueous salt solutions (Merck, Darmstadt, Germany) were buffered with 10 mM MES-KOH to pH 5.5 to pH 6. Control experiments revealed that the pH was stable during the time course of the experiments. The binding components of the binary toxins were reconstituted into the lipid bilayer membranes by adding concentrated solutions to the aqueous phase on one side (the *cis*-side) of a black membrane. The temperature was kept at 20°C throughout. Membrane conductance was measured after application of a fixed membrane potential with a pair of silver/silver chloride electrodes inserted into the aqueous solutions on both sides of the membrane. Membrane current was measured using a homemade current-to-voltage converter combined with a Burr Brown operational amplifier. The amplified signal was monitored on a storage oscilloscope and recorded on a strip chart recorder.

### Binding Experiments

The binding of the His-tagged proteins to the C2II-channel and the binding component PA_63_ was investigated with titration experiments similar to those performed previously to study the binding of 4-aminoquinolones to the C2II- and PA_63_-channels and EF and LF to the PA_63_-channel in single- or multi-channel experiments [38]–[40]. The aqueous phase contained always 150 mM KCl, buffered with 10 mM MES-KOH to pH 5.5 to pH 6. The C2II- and PA_63_-channels were reconstituted into lipid bilayers. About 60 minutes after the addition of either activated C2II or PA_63_ to the *cis*-side of the membrane, the rate of channel insertion in the membranes was very small. Then concentrated solutions of His-tagged proteins were added to the *cis*-side of the membranes while stirring to allow equilibration. The results of the titration experiments, i.e. the blockage of the channels, were analyzed using Langmuir adsorption isotherms [21], [41].
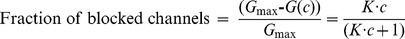
(1)


The Fraction of closed channels as a function of the concentration of the enzymatic components was analyzed using Lineweaver-Burk (double reciprocal) plots.
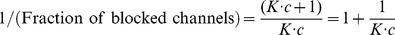
(2)


*K* is the stability constant for binding of the enzymatic components of the binary toxins to the PA_63_- or C2II-channels. The half saturation constant *K_s_* is given by the inverse stability constant 1/*K*.

### Statistical Analysis

When assessing multiple groups, one-way ANOVA was used followed by Bonferroni post hoc test with *p<0.05. Data are presented as means ± SEM. The statistical software used was Prism 5.0 b.

## Results

### Interaction of PA_63_-pores with His_6_-C2I in Artificial Black Lipid Bilayer Membranes

We compared the binding affinity of different proteins with and without a His_6_-tag to the PA_63_- and C2II-channels. Taking into account that positive charges seem to have a huge influence in binding to the PA_63_-pore but only less to the C2II-pore [40], [42], we chose the enzymatic component C2I as the first substrate. In a previous study we could show that it binds to PA_63_-pores and could even be translocated into cells albeit with very low efficiency at high C2I concentration [43]. We now addressed the question, if binding and translocation are enhanced by addition of a His_6_-tag to C2I.

The stability constants *K* (and the half-saturation constant *K_s_*) for the binding of His_6_-C2I to the PA_63_-channel were measured in multichannel experiments, performed as described previously [39]. A receptor is required for the binding and oligomerization of PA_63_ on the surface of mammalian cells [8]. However, this is not necessary for reconstitution of PA_63_-channels in artificial lipid bilayers, where channel formation is obtained under mildly acidic conditions [44]. 60 minutes after the addition of the protein to the *cis*-side of the lipid bilayer, the rate of conductance increase had slowed down considerably at an applied membrane potential of 20 mV. At that time, small amounts of a concentrated protein solution were added to the *cis*-side of the membrane and the PA_63_-induced membrane conductance decreased in a stepwise manner.

**Figure 1:**
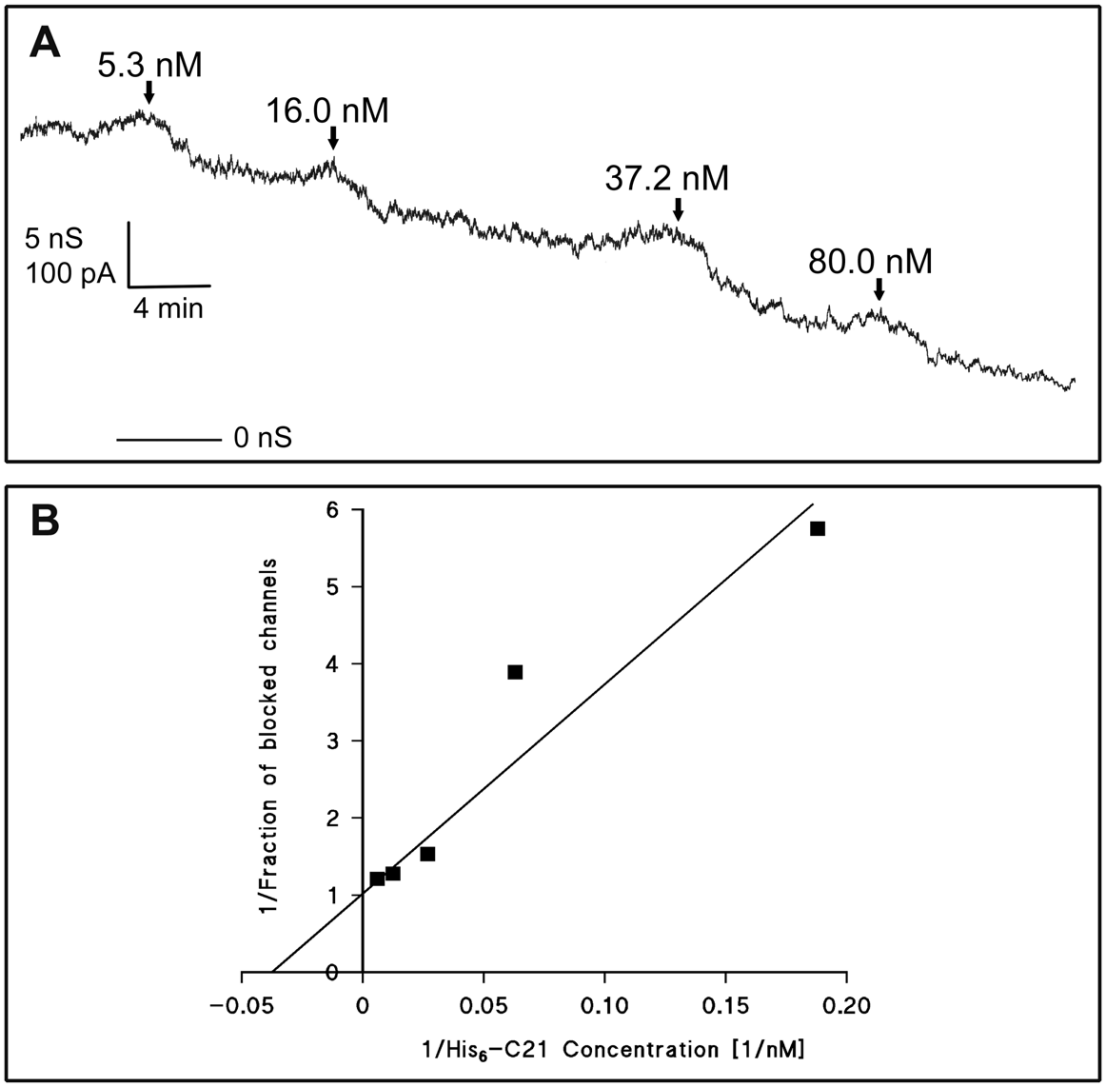
Interaction of C2I with PA_63_ channels. A: Titration of PA_63_ induced membrane conductance with His_6_-C2I. The membrane was painted from 1% (w/v) diphytanoyl phosphatidylcholine dissolved in n-decane. It contained about 300 PA_63_-channels. His_6_-C2I was added at the concentrations shown at the top of the panel to the *cis*-side of the membrane. Finally, about 83% of the PA_63_-channels were blocked. The aqueous phase contained 1 ng/ml activated PA_63_ (added only to the *cis*-side of the membrane), 150 mM KCl, 10 mM MES-KOH pH 6. The temperature was 20°C and the applied voltage was 20 mV. B: Lineweaver-Burk (double reciprocal) plot of the inhibition of the PA_63_-induced membrane conductance by His_6_-C2I using equation (2). The fit was obtained by linear regression of the data points taken from Figure 1A and corresponds to a stability constant *K* for His_6_-C2I binding to PA_63_ of (3.93±0.39)×10^7^ M^−1^ (r = 0.955; half saturation constant *K_s_* = 25 nM).

Figure 1A shows an experiment of this type in which increasing concentrations of His_6_-C2I (arrows) were added to the *cis*-side of a membrane containing about 300 PA_63_-channels. The membrane conductance decreased as a function of the His_6_-C2I concentration. The data of Figure 1A and of similar experiments were analyzed assuming Langmuir isotherms for binding (equation (1)) [39], [41], [45] Lineweaver-Burk (double reciprocal) plots were used to calculate the stability constant *K* for binding as shown in Figure 1B for the data of Figure 1A. The resulting straight corresponded to a stability constant *K* of (3.93±0.39)×10^7^ M^−1^ for His_6_-C2I binding to PA_63_-pores (half saturation constant KS = 25 nM).

At least three individual experiments were used to calculate the half saturation constants *K_S_* of C2I- and His_6_-C2I binding to the PA_63_-channel. The average of the half-saturation constant *K_s_* was 150 nM for C2I, whereas that for His_6_-C2I binding to PA_63_-channels was 16 nM] in 150 mM KCl. This means that the half saturation constant *K_S_* for binding of His_6_-C2I was roughly ten times lower than that for C2I without His_6_-tag (Table 1). Titration experiments with artificial bilayer membranes of the wildtype A-B components C2II and C2I of C2-toxin revealed a half saturation constant *K_S_* of 27 nM. Interestingly, a His_6_-tag attached to the N-terminal end had no obvious effect on binding of C2I to C2II-pores (*K_S_* = 29 nM; Table 1).

**Table 1:** Stability constants *K* and half saturation constants *K_s_* for binding of proteins with and without His_6_-tags to membrane channels formed by anthrax PA_63_ and C2II.

PA_63_		*K* [10^7^ 1/(Ms)]	*K_s_* [nM]		*K* [10^7^ 1/(Ms)]	*K_s_* [nM]	Ratio *K_S_/K_S_* without and with His_6_-tag
**with**	EF*	14.5	6.9	His_6_-EF*	62.5	0.16	43
	LF*	36.2	2.8	His_6_-LF*	550	0.18	16
	C2I	0.68±0.42	150	His_6_-C2I	6.2±4.2	16	9.4
	gpJ	<0.001	>100.000	His_6_-gpJ	20±6.0	5.0	>20,000
	**EDIN**	**0.040**±**0.011**	**2,700**	**His_6_-EDIN**	**0.14**±**0.015**	**700**	**3.9**
**C2II**		***K*** ** [10^7^ 1/(Ms)]**	***K_s_*** ** [nM]**		***K*** ** [10^7^1/(Ms)]**	***K_s_*** ** [nM]**	
**with**	EF**	7.7	13.0	His_6_-EF	5.2±1.6	19	0.68
	LF**	2.0	49.9	His_6_-LF	3.4±1.9	29	1.7
	C2I**	3.7	27.2	His_6_-C2I	3.9±0.52	29	0.94
	gpJ	<0.001	>100,000	His_6_-gpJ	<0.001	>100,000	Not to determine
	EDIN	0.0043±0.0007	23,000	His_6_-EDIN	0.11±0.03	900	26

Stability constants *K* and half saturation constants *K_s_* for the binding of His_6_-tagged and untagged EF, LF, C2I, gpJ or EDIN to PA_63_- or C2II-channels in lipid bilayer membranes. The membranes were painted from 1% (w/v) diphytanoyl phosphatidylcholine dissolved in n-decane. The aqueous phase contained 150 mM KCl, buffered to pH between 5.5 and 6 using 10 mM MES-KOH; T = 20°C. Measurements were performed at a membrane potential of 20 mV. The data represent the means (± SD) of at least three individual titration experiments. *K_S_* is the half saturation constant, i.e. *K_S_* = 1/*K*. Stability constants given in bold were adjusted to the voltage dependent behavior of binding. (* taken from [21] ** taken from [43]).

### Addition of His_6_-tag to C2I Potentiates its Transfer via PA_63_

**Figure 2:**
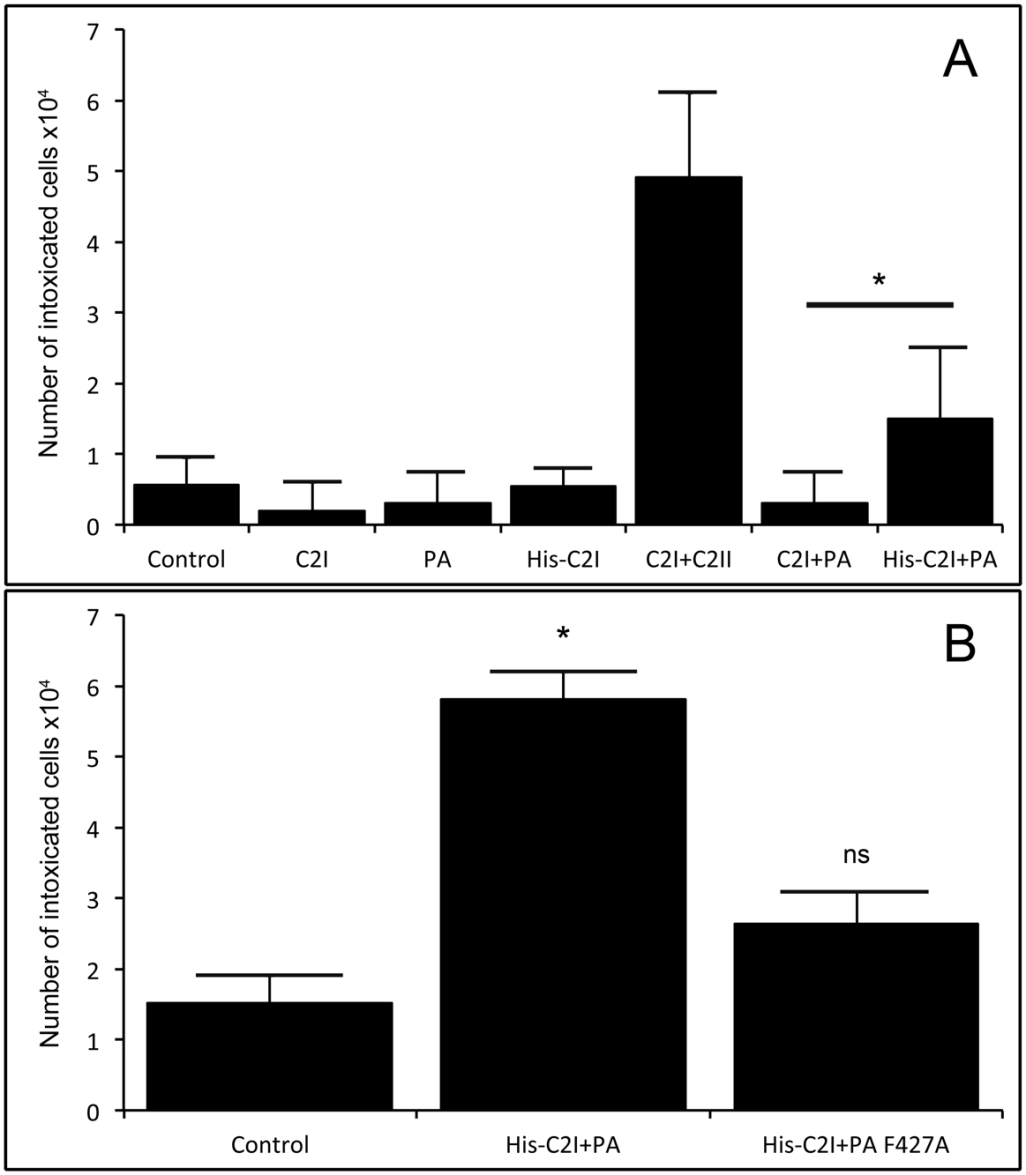
Efficiency of HUVEC intoxication by C2I and His_6_-C2I using PA_63_ compared to PA F427A mutant. HUVECs (5×10^5^ cells/100 mm well) were intoxicated during 24 hours and the number of intoxicated cells (round cells) was assessed by counting floating cells. A: PA_63_ and C2II at 5 µg/ml and C2I and His_6_-C2I at 2 µg/ml. One representative experiment showing mean values of 5 independent counting for each condition. ± SEM *p<0.05 versus control condition. B: PA_63_ and PA F427A at 50 µg/ml and His_6_-C2I at 2 µg/ml. Data correspond to mean values of n = 5 experiments ± SEM, *p<0.05 versus control condition. The control corresponds to conditions without PA_63_. All experiments were performed with the same batch of cells at the same time. The 3-fold increase of toxicity using 10-fold more PA_63_ was repeatedly measured.

In further experiments we tested if addition of His_6_-tag to C2I triggers its entry into cells via PA_63_-channels *in vivo*. C2I acts as an ADP-ribosyltransferase, targeting cellular G-actin [46]. Therefore, successful delivery of this enzymatic component into target cells can be detected by disruption of the cytoskeleton followed by rounding up and detachment of target cells from the extracellular matrix, defined as intoxicated cells [15]. HUVECs were intoxicated with C2I and His_6_-C2I driven by PA_63_, as indicated, and the number of intoxicated cells was directly assessed by counting (Fig. 2A). These results were compared to that of native toxin combination C2I and C2II. We observed a cytotoxic effect with the combination of His_6_-C2I and PA_63_. No effect could be detected for C2I and PA_63_ under the same conditions. The specificity of this internalization was verified by using a mutant of PA_63_: PA F427A. This mutant is competent for receptor binding and internalization, but defective in the pH dependent functions: pore formation and ability to translocate bound ligands [47]. Intoxication of cells with His_6_-C2I and PA F427A did not induce any cellular effect (Fig. 2B). Thus, the increase of affinity between PA_63_ and C2I, upon addition of His_6_-tag to C2I allows His_6_-C2I to efficiently intoxicate cells via PA_63_-channels.

### His_6_-tags do not Facilitate Binding of EF and LF to C2II-channels

To examine whether the N-terminal His_6_-tag of EF and LF have a similar effect on binding kinetics to the C2II-channel, as previously shown for His_6_-EF and His_6_-LF and PA_63_ [21], we omitted the cleavage of the His_6_-tag after the affinity purification and studied binding to C2II-channels. Interestingly, His_6_-EF and His_6_-LF did not exhibit any significant changes of their affinity to C2II-channels as compared to EF and LF (see Table 1). The half saturation constants *K_S_* of the interactions between His_6_-EF and His_6_-LF and the C2II-channels were 19 nM for His_6_-EF and 29 nM for His_6_-LF (Table 1).

### Binding of His_6_-gpJ and gpJ Proteins to PA_63_- and C2II-channels

The His_6_-tag had a remarkable influence on binding of enzymatic components to the PA_63_-channel but not to the C2II-channel. To check if this interaction was specific for the presence of the His_6_-tag we performed titration experiment with a His-tagged protein that is not related to the effectors EF, LF or C2I. gpJ is a 447 amino acids C-terminal fragment of protein J (amino acids 684–1131), which is responsible for binding of bacteriophage Lambda to LamB on the surface of *E. coli* K-12 [31]. His_6_-gpJ exhibited high affinity binding (block) to the PA_63_-channel. The half saturation constant *K_S_* for binding of His_6_-gpJ to PA_63_ was calculated to be 5.0 nM in 150 mM KCl, 10 mM MES-KOH, pH 6.0 (mean of three measurements) (Table 1). Similar experiments with gpJ did not exploit any binding of gpJ to the PA_63_-channel. This implies half saturation constants *K_s_* of gpJ-binding to PA_63_ were much higher than 100 µM. We could not detect any substantial binding of His_6_-gpJ nor of gpJ to the C2II-channel (Table 1). Our results reveal the substantial role of the His_6_-tag at the N-terminal end of C2I and gpJ for their binding to the PA_63_- but not to the C2II-channel.

### Binding of EDIN and His_6_-EDIN to PA_63_- and C2II-channels

Next, we tested whether PA_63_-pores bind and transport EDIN of *Staphylococcus aureus*, as well as His_6_-EDIN. EDIN normally enters host cells inefficiently by nonspecific macropinocytosis and not by delivery systems considered in this study [28]. Previously, it has been shown that LF_1–254_-EDIN can enter cells via PA_63_ [28]. EDIN is a *Staphylococcus aureus* exoenzyme with ADP-ribosylating activity on RhoA. EDIN targets RhoA in cells for inactivation producing actin cable disruption in target cells [34]. Interestingly, we found that PA_63_-pores bound both EDIN and His_6_-EDIN with half saturation constants that were considerably lower than those reported before for the crossing over of the AB_7/8_ types of toxin [43]. The half saturation constant *K_S_* for EDIN binding to PA_63_-channels was on average 2.7 µM in 150 mM KCl, whereas this constant decreased to 0.7 µM for His_6_-EDIN. The results of these experiments are summarized in Table 1 and demonstrate that EDIN without His_6_-tag bound at low transmembrane voltage (5 mV) with a roughly four-fold lower affinity to the PA_63_-channels than His_6_-EDIN. When higher voltages were applied we noticed a remarkable effect of voltage on His_6_-EDIN binding (see below). The affinity of EDIN to the C2II-channels (*K_S_* = 23 µM) was by a factor of about eight-fold lower as compared to binding to the PA_63_-channels. Surprisingly, we observed a considerable effect when the His_6_-tag was attached to the N-terminal end of EDIN. The half saturation constant dropped in this case to 0.9 µM for its binding to C2II-pores (Table 1).

### His_6_-tag Promotes EDIN Internalization via PA_63_-pores

**Figure 3:**
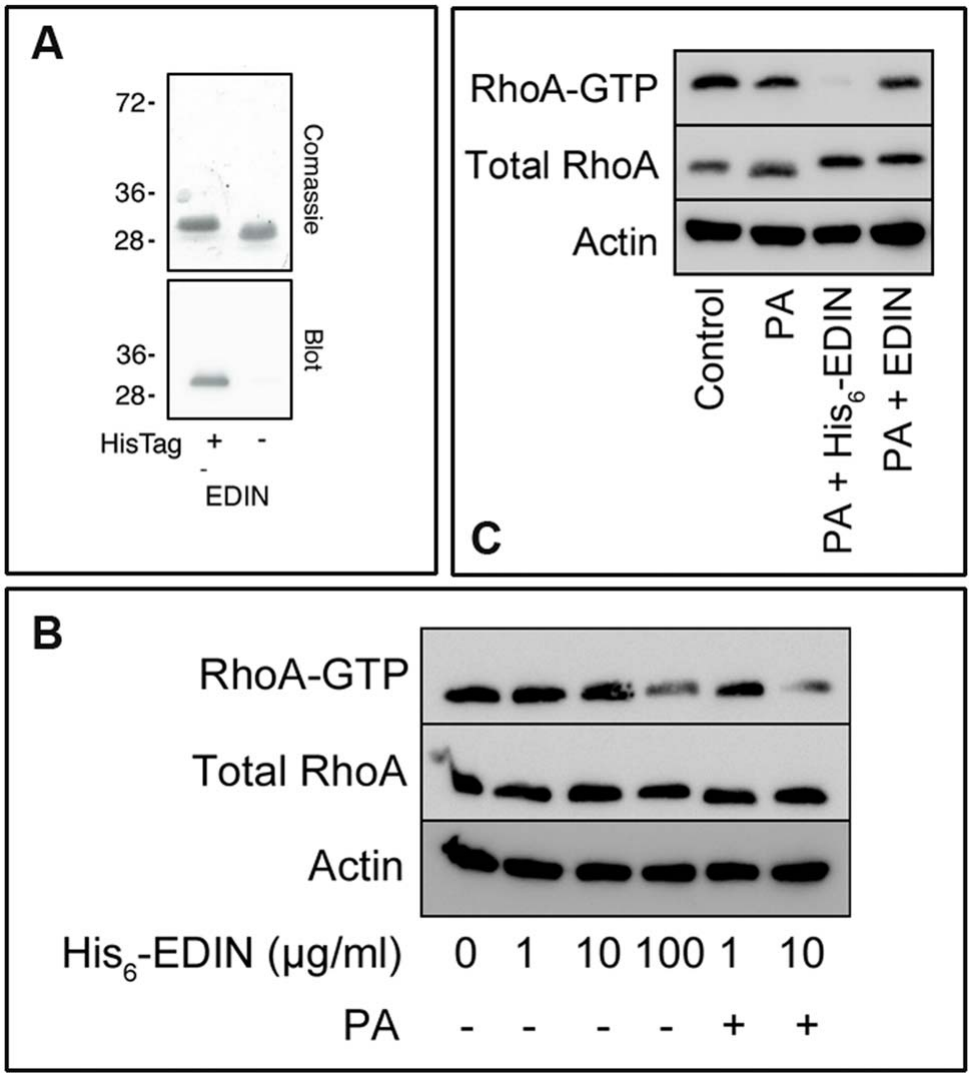
His_6_-tag allows internalization of EDIN in endothelial cells through PA_63_. A: Upper panel: SDS-PAGE of recombinant His_6_-tagged EDIN before (left) and after thrombin treatment (right). Lower panel: immunoblot anti–His_6_-tag on His_6_-tagged EDIN before and after cleavage by thrombin. B, C: Immunoblots showing cellular levels of active RhoA (RhoA-GTP) in HUVECs determined by GST-Rhotekin RBD pulldown (labeled RhoA-GTP). Cellular content of RhoA (Total RhoA) was assessed by anti-RhoA on 2% of total protein extracts. Immunoblot anti-actin antibody exhibits equal protein loading. (B) Cells were intoxicated with different concentrations of His_6_-EDIN (1, 10 and 100 µg/ml) with and without 3 µg/ml of PA_63_, as indicated. (C) Cells were intoxicated with 10 µg/ml His_6_-EDIN, 10 µg/ml EDIN, and 3 µg/ml PA_63_ as indicated.

We next verified the role of His_6_-tag in the uptake of EDIN into cells. After purification the His_6_-tag was cleaved as described in the material and methods section. We verified the cleavage by immunoblotting the purified proteins using an antibody against the His_6_-tag (Fig. 3A). The efficiency of RhoA targeting by EDIN was assessed by GST-Rhotekin pull down of active RhoA (GTP-bound RhoA). No effect was measured on cells challenged with His_6_-EDIN alone up 10 µg/ml (Fig. 3B).

We then intoxicated cells with His_6_-EDIN in the presence and absence of PA_63_. Strikingly, this revealed that the addition of PA_63_ together with His_6_-EDIN (10 µg/ml) increased the capacity of EDIN to intoxicate cells. This led us to decipher the role of His_6_-tag. Cells were intoxicated with PA_63_ together with EDIN or His_6_-EDIN. This clearly established that addition of His_6_-tag to EDIN in presence of PA_63_ produced a 78% decrease of RhoA activation specifically (Fig. 3C). In conclusion, addition of His_6_-tag to EDIN promotes its internalization via PA_63_ for cell intoxication.

### Immunofluorescence Studies of HUVECs and PA_63_ with EDIN and His_6_-EDIN

**Figure 4:**
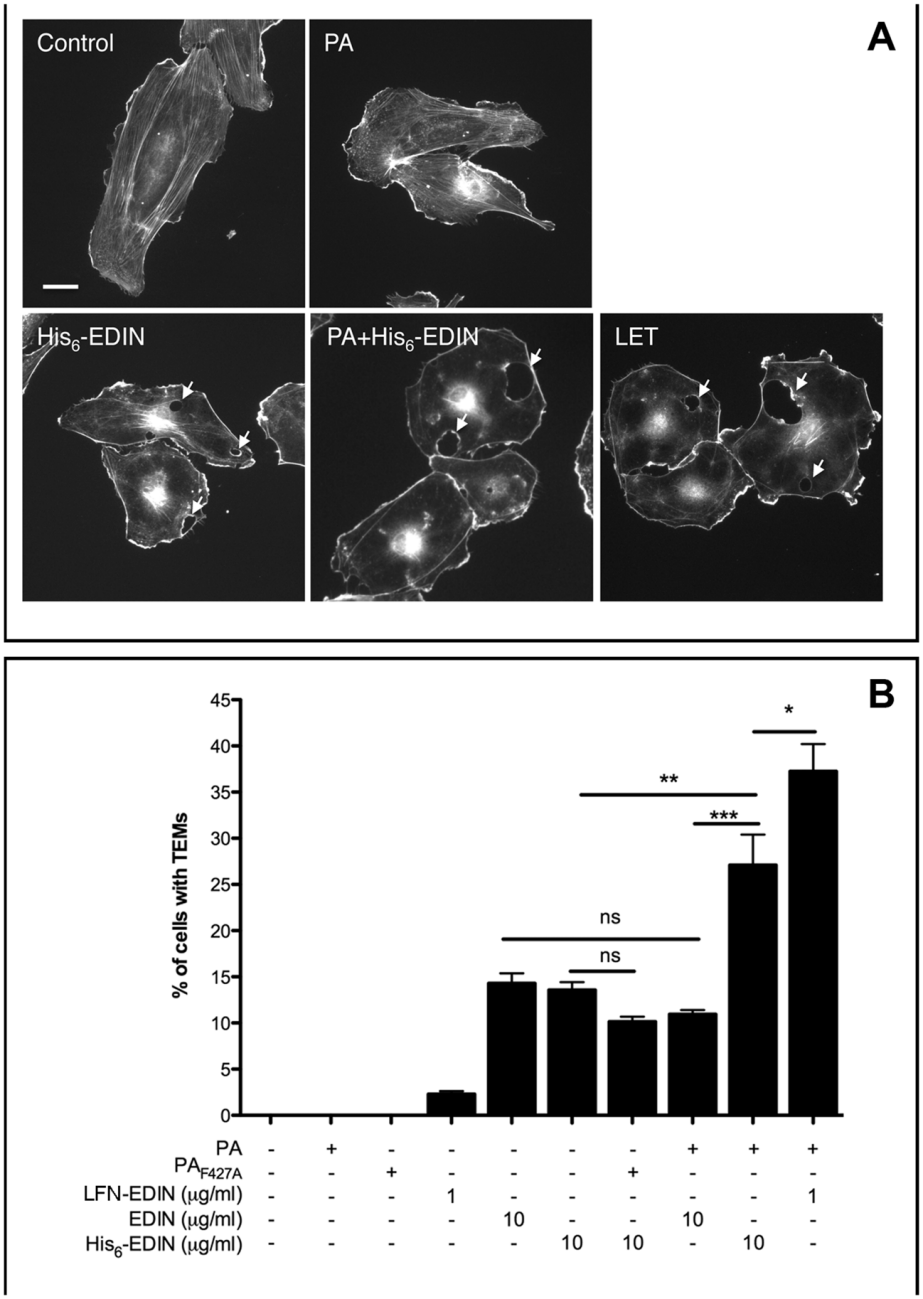
Immunofluorescence studies of HUVECs treated with EDIN and His_6_-EDIN and PA_63_. A: HUVECs were intoxicated for 24 h with a combination of PA_63_ 3 µg/ml, His_6_-EDIN 10 µg/ml and LF_1–254_-EDIN (LFN-EDIN) 1 µg/ml, as indicated. Cells were fixed and actin cytoskeleton was labelled using FITC-conjugated phalloidin. Bar = 10 µm. Arrows indicate transendothelial cell macroaperture tunnels (TEMs, transcellular tunnels). B: Graph shows percentage of cells with toxin-induced transendothelial cell macroaperture tunnels (TEMs, transcellular tunnels). HUVECs were intoxicated for 24 h with a combination of PA_63_ 3 µg/ml, His_6_-EDIN or EDIN 10 µg/ml and LF_1–254_-EDIN (LFN-EDIN) 1 µg/ml, as indicated on the graph legend. Data correspond to means ± SEM (n = 3, 400 cells per condition).

We next analyzed the actin cytoskeleton phenotype of cells intoxicated with PA_63_+His_6_-EDIN and PA_63_+LFN-EDIN, as well as EDIN alone or in combination with PA_63_ (Figure 4A). In all cases we observed that intoxicated cells displayed a disruption of filamentous actin and actin cables, as well as a undergo spreading, as previously described (Figure 4A) [34], [36]. In addition, intoxicated cells displayed large transendothelial cell macroaperture tunnels (TEM) (see Figure 4A). Induction of TEMs results from the dose dependent inhibition of RhoA [34], [36]. We thus further determined the efficiency of cell intoxication, using different combinations of toxin components by measure of the percentage of cells displaying TEMs (Figure 4B). Most importantly, this showed that addition of His_6_-tag to EDIN increases its capacity to intoxicate cells in combination with PA_63_ at a concentration of 10 µg/ml, whereas intoxication of HUVECS with LFN-EDIN saturated already at 1 µg/ml (Figure 4B).

### The Voltage Dependency of PA_63_-channels is Changed when His_6_-EDIN is Bound to the Pore

**Figure 5:**
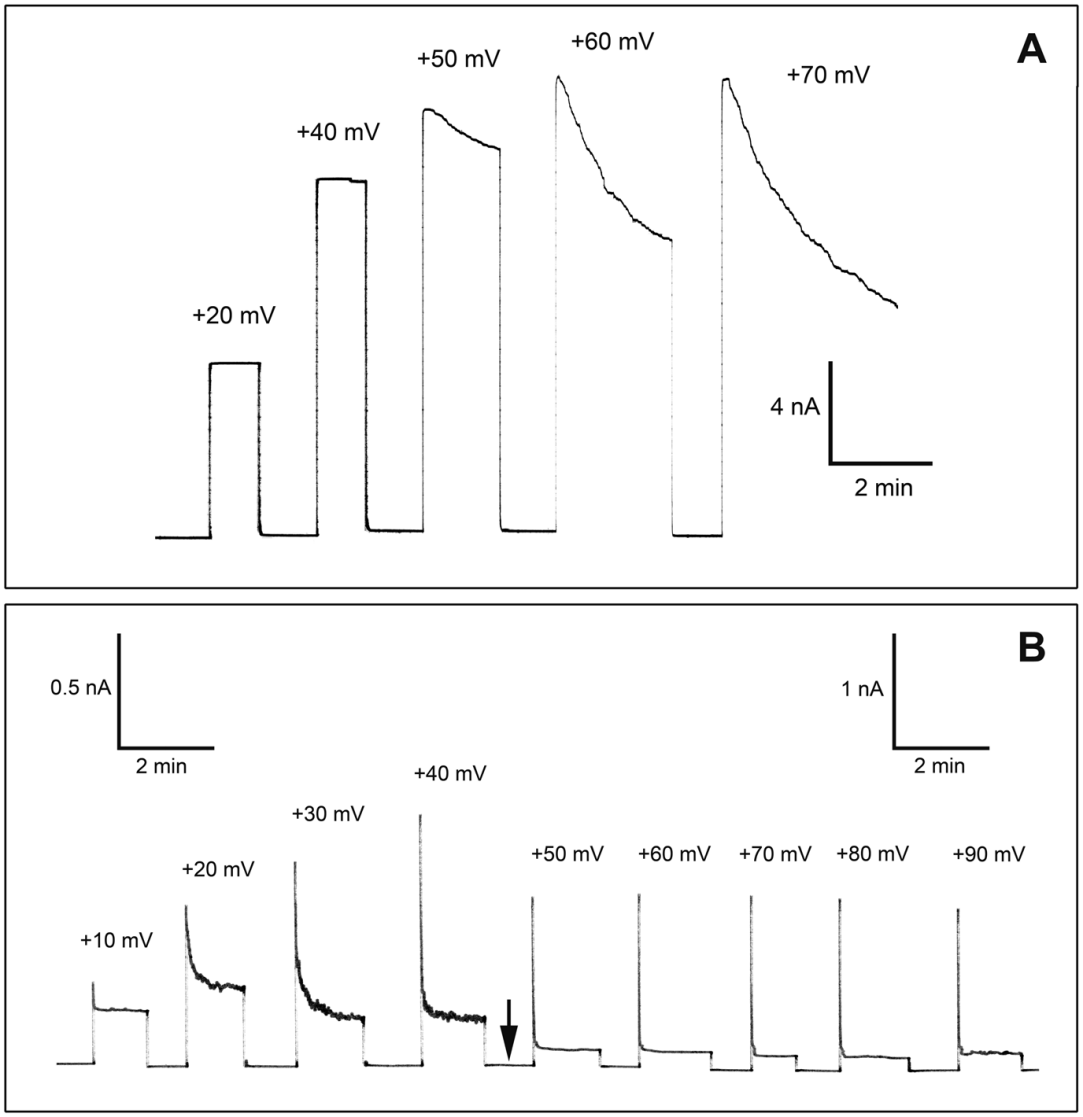
Voltage dependency of PA_63_-channels in the presence of EDIN and His_6_-EDIN. A: Current response of PA_63_-channels in presence of EDIN. Voltage pulses between +20 and +70 mV were applied to a diphytanoyl phosphatidylcholine/n-decane membrane in the presence of PA_63_-pores and EDIN (both added only to the cis side of the membrane). The aqueous phase contained 150 mM KCl, 10 mM MES-KOH, pH 6. The temperature was 20°C. B: Current response of PA_63_ channels in the presence of His_6_-EDIN. Voltage pulses between +10 and +90 mV were applied to a diphytanoyl phosphatidylcholine/n-decane membrane in the presence of PA_63_-pores and His_6_-EDIN (both added only to the cis side of the membrane). The aqueous phase contained 150 mM KCl, 10 mM MES-KOH, pH 6. The temperature was 20°C. Note the change of the scale (Arrow).

PA_63_-channels exhibit a well described voltage dependency [21]. If only added to the cis-side, PA_63_-induced conductivity decreases when applied voltage is higher than +50 mV or lower than −20 mV at the cis-side. It is also known that His_6_-EF bound to the channel changes the voltage dependency [21]. When different potentials were applied to membranes after the titration of PA_63_-pores with EDIN, there was only little change in voltage dependency of the channel (Fig. 5A). On the other hand, His_6_-EDIN bound to PA_63_-channels induced dramatic responses even at low positive voltages (Fig. 5B). Starting at +10 mV, the conductivity decreased exponentially immediately after the onset of the voltage with a voltage-dependent exponential relaxation time. Its time constant decreased with higher positive potentials at the cis-side (negative at the trans-side). This result indicated that channels, which were not blocked before by His_6_-EDIN at low voltage bound this compound and closed as a result of the higher voltage. This result suggested an increase of the stability constant of binding up to very high voltages an effect that has already been observed with full length EF [21].

**Figure 6:**
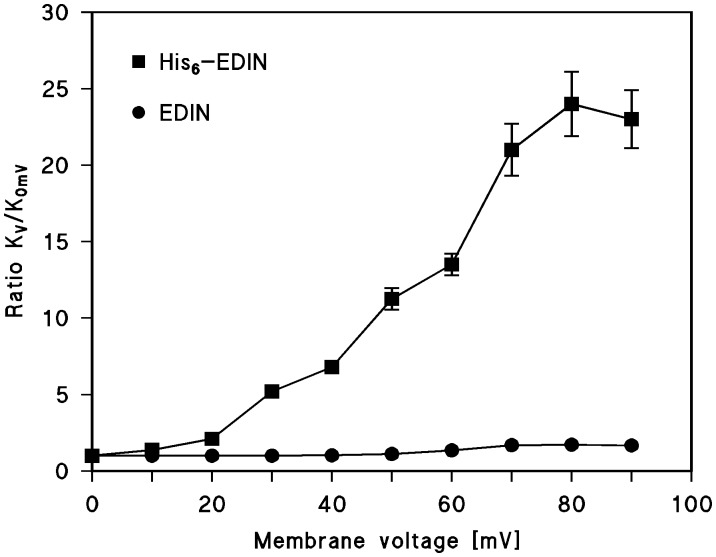
Correlation of affinity constant *K* and voltage dependence of PA_63_-channels in presence of EDIN and His_6_-EDIN. The stability constants of EDIN and His6-EDIN binding to the PA_63_-channel are given as a function of the applied membrane potential taken from experiments similar to that shown in Fig. 5 A/B. Means of three experiments are shown.

The increase of the stability constant for binding could be calculated from the data of Figures 5A and 5B and similar experiments by dividing the initial current (which was a linear function of voltage) by the stationary current after the exponential relaxation and multiplying the ratio with the stability constant derived at 5 mV. Figure 6 summarizes the effect of the positive membrane potential on the stability constant K for EDIN and His_6_-EDIN binding as a function of the voltage. Starting already with −10 mV at the trans-side the stability constant K for His_6_-EDIN binding started to increase and reached with about 60 to 70 mV a maximum. At that voltage K was roughly 25 times greater than at 5 mV. For higher voltages the stability constant saturated probably because of secondary effects of the high voltage on the PA_63-_channel or on His_6_-EDIN binding. Figure 6 shows also the effect of the positive membrane potential at the cis-side on the stability constant K for EDIN binding to PA_63_-pores as a function of the voltage. Interestingly, EDIN binding was only little affected by voltage as Figure 6 clearly indicated.

## Discussion

### His_6_-tag Addition to Several Bacterial Factors Increased the Protein Binding Affinity to PA_63_- but not to C2II-channels

Recent studies demonstrated that negatively charged amino acids in the vestibule of the PA_63_-channel play a crucial role in binding of effector molecules [40], [42]. Thus, it is possible that a His_6_-tag, which adds positive charges under mildly acidic conditions to the N-terminal end of His_6_-EF and His_6_-LF affects binding and transport. This has indeed been shown for the native combinations of EF+PA_63_ or LF+PA_63_ and the potential ion-ion interaction discussed with EF_N_ [21], [39], [48], [49]. Recently, we could show that C2I binds to PA_63_ and may even be transported into target cells albeit at high PA_63_ concentration and with very low efficiency compared with the native combination of C2I with C2II [43]. Here we studied the cross reactivity of anthrax- and C2-toxin in more detail and found a strong relation between binding affinity and the presence of a His_6_-tag at the N-terminal end of the enzymatic components. The addition of positive charges at the N-terminal end of C2I (due to the partially charged histidines) enhanced binding to and translocation into target cells via PA_63_-pores and agreed very well with the findings previously reported for His_6_-tags attached to EF and to LF [21], [39]. Binding to PA_63_-channels was found to be strongly enhanced for the three enzymatic components EF, LF and C2I when they contained a His_6_-tag at the N–terminal end.

Interestingly, we did not observe major effects if these His_6_-tagged proteins were combined with C2II-channels. The results of binding experiments with His_6_-EF and His_6_-LF to C2II-channels suggested that the increased positive charge at the N-terminal end, due to the partially charged histidines, did not increase the binding of these enzymatic components to the C2II-channels. These results definitely imply that binding of the enzymatic components to PA_63_-channels occurs in a different way than binding to C2II-channels.

To gain deeper insight in the influence of N-terminal His_6_-tags on binding of proteins to PA_63_-channels we choose a protein, gpJ, that was not related to any of the enzymatic components used in this study [33]. As expected, we did not observe any binding of gpJ to PA_63_- or C2II-channels (*K_S_* >100 µM). However, binding was observed when a His_6_-tag was attached to the N-terminal end of gpJ. This protein had a half-saturation constant for binding to the PA_63_-channels of 5 nM, which suggested that the affinity of His_6_-gpJ to PA_63_-channels was almost the same as that of LF and EF [21], [39]. This means that the affinity increase is mainly determined by the positive charges of the His_6_-tag. It is interesting to note that His_6_-gpJ did accordingly not interact with C2II-channels; revealing again a somewhat different process for binding of His_6_-tagged proteins to PA_63_-channels than to C2II-channels. However, it is not clear if His_6_-gpJ could also be imported via PA_63_ into target cells because this protein has no intracellular enzymatic activity [320].

### Influence of the His_6_-tag on Uptake of EF, LF and C2I into Cells

The binding step is a prerequisite, but not sufficient for the delivery of enzymatic subunits into target cells. Thus, in order to complement the results of binding studies we went on to investigate the translocation by analyzing the enzymatic activity in a cellular system. We verified that a His_6_-tag attached to the N-terminal end of C2I increased its transport by PA_63_-channels, which correlates with the difference of 10-fold measured between the stability constants for binding of C2I and His_6_-C2I. Some difference in transport was observed by using EF or LF with or without a His_6_-tag in combination with PA_63_-channels. In particular, we could demonstrate that addition of a His_6_-tag promoted uptake of LT (PA + His_6_-LF) into HUVECs (data not shown). This result is in agreement with the increased affinity of EF and LF to PA_63_-heptamer/octamerss when they contain a His_6_-tag at the N-terminus [21], [23].

Although the binding of EF_N_ and LF_N_ (truncated forms of EF and LF) to the PA_63_–channel is substantially weaker as compared to wild-type enzymatic components [42], these proteins interact with high affinity with the PA_63_-channels and are accordingly transported into the cell [48], [50], [51]. Similarly, short stretches of positively charged amino acids attached to the N-terminal end of foreign proteins can lead to a PA_63_-dependent delivery as it has been demonstrated for the addition of polycationic peptides to the N-terminus of the enzymatic A chain of diphtheria toxin (DTA; residues 1–193) or for LF_1–254_-EDIN [19], [28].

### Bound His_6_-EDIN or EDIN Causes a Difference in Voltage-dependency of PA_63_-pores

Experiments with EDIN of *S. aureus* were performed to gain deeper insight in the binding and translocation processes through PA_63_-channels and its His_6_-tag dependence. Surprisingly, black lipid bilayer experiments displayed that not only His_6_-EDIN but also EDIN itself bound to PA_63_-channels. The affinity of EDIN and His_6_-EDIN to the PA_63_-channels was in the same range at low trans-membrane potentials because His_6_-EDIN exhibited only a three times higher affinity for binding to the PA_63_-channels than EDIN. Under normal conditions the PA_63_-channels only close for higher negative voltages applied to the cis-side [21]. For positive potential the channels are open and do not show a voltage-dependent closure until 100–150 mV [21]. However, His_6_-EDIN binding to the PA_63_-channels showed an extremely high voltage-dependence when the voltage was positive at the cis-side of the membrane indicating that the potential pulled His_6_-EDIN into the channels. As a result the stability constant for binding of His_6_-EDIN to the PA_63_-channels increased at voltages of +70 mV at the cis-side (corresponding to −70 mV at the trans-side) by a factor of roughly 25 as compared to zero voltage. Bound EDIN displayed an only minor voltage-dependence. This means that the His_6_-tag is responsible for the binding EDIN and gpJ to the PA_63_-channels. Binding is essential for translocation because it is the first step of the whole process (see below).

### The PA_63_-channel Transports His_6_-C2I and His_6_-EDIN into the HUVECs

EDIN uptake into target cells can easily be detected because it decreases RhoA activity. Import of EDIN via PA_63_-channels could not be observed. Import was however, possible when EDIN contained a His_6_-tag. This finding demonstrated that His_6_-tag itself provides the ability for proteins to be transported into cells through PA_63_-pores. This effect was presumably promoted by the voltage-dependence of His_6_-EDIN binding to the PA_63_-channels. Biological membranes are polarized to about −60 mV to −70 mV (inside negative). This may explain the much higher effect of His_6_-EDIN compared to EDIN on cells described above. In any case it clearly indicates the potentiating effect of a His_6_-tag and applied voltage on binding and translocation of protein molecules to PA_63_-channels. Summarizing the results, there definitely exists a difference in the binding and translocation mechanism between the two very homologous binding components PA_63_ and C2II of anthrax- and C2-toxin. Obviously, this distinction is induced by unequal binding surroundings inside the head region of the two protein channels.

The amino acids responsible for binding within the N-terminal end of the enzymatic components are still a matter of debate, although there is clear evidence that positively charged amino acids are involved in binding, forming salt bridges between the enzymatic components and the heptamers/octamers. In a recent study it has been shown that besides the Phi-clamp also the such-called alpha-clamp is also involved in effector binding, unfolding and translocation in combination with PA_63_ [26], [27]. This alpha-clamp is composed of hydrophobic and aromatic residues, such as F202 and P205 and forms a deep amphipathic cleft on the surface of the PA_63_ oligomer [26], [27]. It is also possible that R178 contributes to effector binding but not to translocation. However, the alpha-clamp does not seem to be very specific because of its broad substrate specificity and non-specific polypeptide binding activity [26]. It is noteworthy that amino acids of the alpha-clamp do not appear to be preserved in C2II because PA R178, PA F202 and PA P205 correspond to C2II T169, C2II W193 and C2II K196 [26], [27]. This could mean that the design of the alpha-clamp in C2II if it existed has a different structure or is absent.

The positively charged N-termini of the enzymes play presumably a crucial role, because quaternary ammonium ions and 4-aminoquinolones show PA_63_ and C2II channel block in lipid bilayer experiments [38], [40], [44], [52]. Both channels show a high selectivity for cations, i.e. cations have a strong influence on the single channel conductance as compared to anions [53], [54]. This means that negative charged amino acids play a crucial role in the binding and constriction region of the PA_63_-channels, where they form two rings of seven putative negatively charged amino acids in the vestibule of this pore (E398 and D426). Similarly, the channel lumen contains additional three rings of seven possibly negatively charged groups (E302, E308 and D315). Some of these charges cannot be found in the C2II-channel lumen, resulting in minor effects of His_6_-tag on binding and transport. However, transport into cells seems to be possible with C2II-pores and when N-terminal parts of C2I are coupled to foreign proteins [1], [55], [56]. The most interesting result of this study was that we could use the anthrax PA_63_-channels to deliver into cells a polypeptide completely unrelated to the AB_7/8_-type of toxins. In fact, we here provide evidence that the His_6_-tag addition on EDIN allows its entry in target cells, in a PA_63_-dependent manner. On the other hand, we would like to stress the point that the natural uptake of EDIN occurs very slowly at very high EDIN concentration (100 µg/ml: see Figure 3C). Nevertheless, here our data support the idea that it seems possible to design a very simple transportation system using His_6_-tags on proteins unrelated to the AB_7/8_-family and PA_63_-channels for biological purpose.

## References

[1] Barth H, Aktories K, Popoff MR, Stiles BG (2004) Binary bacterial toxins: biochemistry, biology, and applications of common Clostridium and Bacillus proteins. Microbiol Mol Biol Rev 68: 373–402. 15353562 10.1128/MMBR.68.3.373-402.2004 PMC515256

[2] Miller CJ, Elliott JL, Collier RJ (1999) Anthrax protective antigen: prepore-to-pore conversion. Biochemistry 38: 10432–10441. 10441138 10.1021/bi990792d

[3] Abrami L, Lindsay M, Parton RG, Leppla SH, van der Goot FG (2004) Membrane insertion of anthrax protective antigen and cytoplasmic delivery of lethal factor occur at different stages of the endocytic pathway. J Cell Biol 166: 645–651. 15337774 10.1083/jcb.200312072 PMC2172425

[4] Petosa C, Collier RJ, Klimpel KR, Leppla SH, Liddington RC (1997) Crystal structure of the anthrax toxin protective antigen. Nature 385: 833–838. 9039918 10.1038/385833a0

[5] Kintzer AF, Thoren KL, Sterling HJ, Dong KC, Feld GK, et al (2009) The protective antigen component of anthrax toxin forms functional octameric complexes. J Mol Biol. 392: 614–29. 10.1016/j.jmb.2009.07.037 PMC2742380 19627991

[6] Mock M, Fouet A (2001) Anthrax. Annu Rev Microbiol 55: 647–671. 11544370 10.1146/annurev.micro.55.1.647

[7] Ascenzi P, Visca P, Ippolito G, Spallarossa A, Bolognesi M, et al (2002) Anthrax toxin: a tripartite lethal combination. FEBS Lett 531: 384–388. 12435580 10.1016/s0014-5793(02)03609-8

[8] Young JA, Collier RJ (2007) Anthrax toxin: receptor binding, internalization, pore formation, and translocation. Annu Rev Biochem 76: 243–265. 17335404 10.1146/annurev.biochem.75.103004.142728

[9] Turk BE (2007) Manipulation of host signalling pathways by anthrax toxins. Biochem J 402: 405–417. 17313374 10.1042/BJ20061891

[10] Aktories K, Barth H (2004) The actin-ADP-ribosylating Clostridium botulinum C2 toxin. Anaerobe 10: 101–105. 16701506 10.1016/j.anaerobe.2003.10.003

[11] Aktories K, Wilde C, Vogelsgesang M (2004) Rho-modifying C3-like ADP-ribosyltransferases. Rev Physiol Biochem Pharmacol 152: 1–22. 15372308 10.1007/s10254-004-0034-4

[12] Boquet P, Lemichez E (2003) Bacterial virulence factors targeting Rho GTPases: parasitism or symbiosis? Trends Cell Biol 13: 238–246. 12742167 10.1016/s0962-8924(03)00037-0

[13] Barth H, Blocker D, Behlke J, Bergsma-Schutter W, Brisson A, et al (2000) Cellular uptake of Clostridium botulinum C2 toxin requires oligomerization and acidification. J Biol Chem 275: 18704–18711. 10749859 10.1074/jbc.M000596200

[14] Blocker D, Barth H, Maier E, Benz R, Barbieri JT, et al (2000) The C terminus of component C2II of Clostridium botulinum C2 toxin is essential for receptor binding. Infect Immun 68: 4566–4573. 10899856 10.1128/iai.68.8.4566-4573.2000 PMC98375

[15] Blocker D, Pohlmann K, Haug G, Bachmeyer C, Benz R, et al (2003) Clostridium botulinum C2 toxin: low pH-induced pore formation is required for translocation of the enzyme component C2I into the cytosol of host cells. J Biol Chem 278: 37360–37367. 12869543 10.1074/jbc.M305849200

[16] Considine RV, Simpson LL (1991) Cellular and molecular actions of binary toxins possessing ADP-ribosyltransferase activity. Toxicon 29: 913–936. 1949064 10.1016/0041-0101(91)90076-4

[17] Wei W, Lu Q, Chaudry GJ, Leppla SH, Cohen SN (2006) The LDL receptor-related protein LRP6 mediates internalization and lethality of anthrax toxin. Cell 124: 1141–1154. 16564009 10.1016/j.cell.2005.12.045

[18] Leppla SH, Arora N, Varughese M (1999) Anthrax toxin fusion proteins for intracellular delivery of macromolecules. J Appl Microbiol 87: 284. 10475968 10.1046/j.1365-2672.1999.00890.x

[19] Blanke SR, Milne JC, Benson EL, Collier RJ (1996) Fused polycationic peptide mediates delivery of diphtheria toxin A chain to the cytosol in the presence of anthrax protective antigen. Proc Natl Acad Sci U S A 93: 8437–8442. 8710889 10.1073/pnas.93.16.8437 PMC38689

[20] Schleberger C, Hochmann H, Barth H, Aktories K, Schulz GE (2006) Structure and action of the binary C2 toxin from Clostridium botulinum. J Mol Biol 364: 705–715. 17027031 10.1016/j.jmb.2006.09.002

[21] Neumeyer T, Tonello F, Dal Molin F, Schiffler B, Benz R (2006) Anthrax edema factor, voltage-dependent binding to the protective antigen ion channel and comparison to LF binding. J Biol Chem 281: 32335–32343. 16954207 10.1074/jbc.M606552200

[22] Krantz BA, Melnyk RA, Zhang S, Juris SJ, Lacy DB, et al (2005) A phenylalanine clamp catalyzes protein translocation through the anthrax toxin pore. Science 309: 777–781. 16051798 10.1126/science.1113380 PMC1815389

[23] Melnyk RA, Collier RJ (2006) A loop network within the anthrax toxin pore positions the phenylalanine clamp in an active conformation. Proc Natl Acad Sci U S A 103: 9802–9807. 16785422 10.1073/pnas.0604000103 PMC1479862

[24] Neumeyer T, Schiffler B, Maier E, Lang AE, Aktories K, et al (2008) Clostridium botulinum C2 toxin. Identification of the binding site for chloroquine and related compounds and influence of the binding site on properties of the C2II channel. J Biol Chem 283: 3904–3914. 18077455 10.1074/jbc.M709807200

[25] Krantz BA, Trivedi AD, Cunningham K, Christensen KA, Collier RJ (2004) Acid-induced unfolding of the amino-terminal domains of the lethal and edema factors of anthrax toxin. J Mol Biol 344: 739–756. 15533442 10.1016/j.jmb.2004.09.067

[26] Feld GK, Thoren KL, Kintzer AF, Sterling HJ, Tang II, et al (2010) Structural basis for the unfolding of anthrax lethal factor by protective antigen oligomers. Nature Struct. Mol. Biol. 17: 1383–1791. 10.1038/nsmb.1923 PMC3133606 21037566

[27] Brown MJ, Thoren KL, Krantz BA (2011) Charge requirements for proton gradient-driven translocation of anthrax toxin. J Biol Chem. 286: 23189–23199. 10.1074/jbc.M111.231167 PMC3123086 21507946

[28] Rolando M, Munro P, Stefani C, Auberger P, Flatau G, et al (2009) Injection of Staphylococcus aureus EDIN by the Bacillus anthracis protective antigen machinery induces vascular permeability. Infect Immun 77: 3596–3601. 19546197 10.1128/IAI.00186-09 PMC2738014

[29] Sellman BR, Nassi S, Collier RJ (2001) Point mutations in anthrax protective antigen that block translocation. J Biol Chem 276: 8371–8376. 11113126 10.1074/jbc.M008309200

[30] Cataldi A, Labruyere E, Mock M (1990) Construction and characterization of a protective antigen-deficient Bacillus anthracis strain. Mol Microbiol 4: 1111–1117. 2122174 10.1111/j.1365-2958.1990.tb00685.x

[31] Tonello F, Naletto L, Romanello V, Dal Molin F, Montecucco C (2004) Tyrosine-728 and glutamic acid-735 are essential for the metalloproteolytic activity of the lethal factor of Bacillus anthracis. Biochem Biophys Res Commun 313: 496–502. 14697216 10.1016/j.bbrc.2003.11.134

[32] Wang J, Hofnung M, Charbit A (2000) The C-terminal portion of the tail fiber protein of bacteriophage lambda is responsible for binding to LamB, its receptor at the surface of Escherichia coli K-12. J Bacteriol 182: 508–512. 10629200 10.1128/jb.182.2.508-512.2000 PMC94303

[33] Berkane E, Orlik F, Stegmeier JF, Charbit A, Winterhalter M, et al (2006) Interaction of bacteriophage lambda with its cell surface receptor: an in vitro study of binding of the viral tail protein gpJ to LamB (Maltoporin). Biochemistry 45: 2708–2720. 16489764 10.1021/bi051800v

[34] Boyer L, Doye A, Rolando M, Flatau G, Munro P, et al (2006) Induction of transient macroapertures in endothelial cells through RhoA inhibition by Staphylococcus aureus factors. J Cell Biol 173: 809–819. 16754962 10.1083/jcb.200509009 PMC2063895

[35] Doye A, Boyer L, Mettouchi A, Lemichez E (2006) Ubiquitin-mediated proteasomal degradation of Rho proteins by the CNF1 toxin. Methods Enzymol 406: 447–456. 16472677 10.1016/S0076-6879(06)06033-2

[36] Maddugoda MP, Stefani C, Gonzalez-Rodriguez D, Saarikangas J, Torrino S, et al (2011) cAMP signaling by anthrax edema toxin induces transendothelial cell tunnels, which are resealed by MIM via Arp2/3-driven actin polymerization. Cell Host Microbe 10: 464–474. 22100162 10.1016/j.chom.2011.09.014

[37] Benz R, Janko K, Boos W, Lauger P (1978) Formation of large, ion-permeable membrane channels by the matrix protein (porin) of Escherichia coli. Biochim Biophys Acta 511: 305–319. 356882 10.1016/0005-2736(78)90269-9

[38] Bachmeyer C, Orlik F, Barth H, Aktories K, Benz R (2003) Mechanism of C2-toxin inhibition by fluphenazine and related compounds: investigation of their binding kinetics to the C2II-channel using the current noise analysis. J Mol Biol 333: 527–540. 14556742 10.1016/j.jmb.2003.08.044

[39] Neumeyer T, Tonello F, Dal Molin F, Schiffler B, Orlik F, et al (2006) Anthrax lethal factor (LF) mediated block of the anthrax protective antigen (PA) ion channel: effect of ionic strength and voltage. Biochemistry 45: 3060–3068. 16503661 10.1021/bi0524316

[40] Orlik F, Schiffler B, Benz R (2005) Anthrax toxin protective antigen: inhibition of channel function by chloroquine and related compounds and study of binding kinetics using the current noise analysis. Biophys J 88: 1715–1724. 15596516 10.1529/biophysj.104.050336 PMC1305228

[41] Benz R, Schmid A, Vos-Scheperkeuter GH (1987) Mechanism of sugar transport through the sugar-specific LamB channel of Escherichia coli outer membrane. J Membr Biol 100: 21–29. 3323520 10.1007/BF02209137

[42] Leuber M, Kronhardt A, Tonello F, Dal Molin F, Benz R (2008) Binding of N-terminal fragments of anthrax edema factor (EF(N)) and lethal factor (LF(N)) to the protective antigen pore. Biochim Biophys Acta 1778: 1436–1443. 18243126 10.1016/j.bbamem.2008.01.007

[43] Kronhardt A, Rolando M, Beitzinger C, Stefani C, Leuber M, et al (2011) Cross-reactivity of anthrax and C2 toxin: protective antigen promotes the uptake of botulinum C2I toxin into human endothelial cells. PLoS One. 2011 6(8): e23133. 10.1371/journal.pone.0023133 PMC3151279 21850257

[44] Finkelstein A (1994) The channel formed in planar lipid bilayers by the protective antigen component of anthrax toxin. Toxicology 87: 29–41. 7512762 10.1016/0300-483x(94)90153-8

[45] Benz R, Schmid A, Nakae T, Vos-Scheperkeuter GH (1986) Pore formation by LamB of Escherichia coli in lipid bilayer membranes. J Bacteriol 165: 978–986. 2419312 10.1128/jb.165.3.978-986.1986 PMC214525

[46] Aktories K, Barmann M, Ohishi I, Tsuyama S, Jakobs KH, et al (1986) Botulinum C2 toxin ADP-ribosylates actin. Nature 322: 390–392. 3736664 10.1038/322390a0

[47] Sun J, Lang AE, Aktories K, Collier RJ (2008) Phenylalanine-427 of anthrax protective antigen functions in both pore formation and protein translocation. Proc Natl Acad Sci U S A 105: 4346–4351. 18334631 10.1073/pnas.0800701105 PMC2393744

[48] Zhang S, Finkelstein A, Collier RJ (2004) Evidence that translocation of anthrax toxin's lethal factor is initiated by entry of its N terminus into the protective antigen channel. Proc Natl Acad Sci U S A 101: 16756–16761. 15548616 10.1073/pnas.0405754101 PMC534726

[49] Zhang S, Cunningham K, Collier RJ (2004) Anthrax protective antigen: efficiency of translocation is independent of the number of ligands bound to the prepore. Biochemistry 43: 6339–6343. 15147218 10.1021/bi049794a

[50] Mogridge J, Cunningham K, Collier RJ (2002) Stoichiometry of anthrax toxin complexes. Biochemistry 41: 1079–1082. 11790132 10.1021/bi015860m

[51] Elliott JL, Mogridge J, Collier RJ (2000) A quantitative study of the interactions of Bacillus anthracis edema factor and lethal factor with activated protective antigen. Biochemistry 39: 6706–6713. 10828989 10.1021/bi000310u

[52] Blaustein RO, Lea EJ, Finkelstein A (1990) Voltage-dependent block of anthrax toxin channels in planar phospholipid bilayer membranes by symmetric tetraalkylammonium ions. Single-channel analysis. J Gen Physiol 96: 921–942. 1704046 10.1085/jgp.96.5.921 PMC2229019

[53] Blaustein RO, Koehler TM, Collier RJ, Finkelstein A (1989) Anthrax toxin: channel-forming activity of protective antigen in planar phospholipid bilayers. Proc Natl Acad Sci U S A 86: 2209–2213. 2467303 10.1073/pnas.86.7.2209 PMC286881

[54] Schmid A, Benz R, Just I, Aktories K (1994) Interaction of Clostridium botulinum C2 toxin with lipid bilayer membranes. Formation of cation-selective channels and inhibition of channel function by chloroquine. J Biol Chem 269: 16706–16711. 7515883

[55] Barth H, Blocker D, Aktories K (2002) The uptake machinery of clostridial actin ADP-ribosylating toxins–a cell delivery system for fusion proteins and polypeptide drugs. Naunyn Schmiedebergs Arch Pharmacol 366: 501–512. 12444490 10.1007/s00210-002-0626-y

[56] Barth H, Roebling R, Fritz M, Aktories K (2002) The binary Clostridium botulinum C2 toxin as a protein delivery system: identification of the minimal protein region necessary for interaction of toxin components. J Biol Chem 277: 5074–5081. 11741886 10.1074/jbc.M109167200

